# Arabidopsis *CPR5* Independently Regulates Seed Germination and Postgermination Arrest of Development through LOX Pathway and ABA Signaling

**DOI:** 10.1371/journal.pone.0019406

**Published:** 2011-04-27

**Authors:** Guilan Gao, Shengchun Zhang, Chengfeng Wang, Xiang Yang, Yaqin Wang, Xiaojun Su, Jinju Du, Chengwei Yang

**Affiliations:** 1 Guangdong Key Lab of Biotechnology for Plant Development, College of Life Science, South China Normal University, Guangzhou, China; 2 Key Laboratory for Crop Germplasm Innovation and Utilization of Hunan Province, Hunan Agricultural University, Changsha, China; Iowa State University, United States of America

## Abstract

The phytohormone abscisic acid (ABA) and the lipoxygenases (LOXs) pathway play important roles in seed germination and seedling growth and development. Here, we reported on the functional characterization of *Arabidopsis CPR5* in the ABA signaling and LOX pathways. The *cpr5* mutant was hypersensitive to ABA in the seed germination, cotyledon greening and root growth, whereas transgenic plants overexpressing *CPR5* were insensitive. Genetic analysis demonstrated that *CPR5* gene may be located downstream of the *ABI1* in the ABA signaling pathway. However, the *cpr5* mutant showed an ABA independent drought-resistant phenotype. It was also found that the *cpr5* mutant was hypersensitive to NDGA and NDGA treatment aggravated the ABA-induced delay in the seed germination and cotyledon greening. Taken together, these results suggest that the *CPR5* plays a regulatory role in the regulation of seed germination and early seedling growth through ABA and LOX pathways independently.

## Introduction

Seed germination is the first adaptive decision in the developments of many land plants. Advances in genetics and molecular physiology have taught us much about the control of germination by the phytohormone abscisic acid (ABA) using the model plant *Arabidopsis thaliana*. For example, ABA helps maintain seed dormancy to ensure that seed germinate under favorable conditions. Immediately after germination, ABA may inhibit the establishment and subsequent development of young seedlings, with this post-germinative arrest representing an early developmental checkpoint to slow seedling growth until better conditions arise [1]–[3].

The phytohormone abscisic acid (ABA) is well known for its regulatory roles in integrating environmental constraints with the developmental programs of plant [2]–[10]. ABA-regulated processes of plant development are generally divided into two broad and overlapping categories: ABA signaling in seeds (maintenance of seed dormancy and control of early seedling development) and ABA signaling in guard cells of more mature plants [11]. Molecular genetics studies have significantly advanced our understanding on the molecular basis of ABA signaling in seeds and seedlings. Notably, through the characterizations of a series of ABA-insensitive mutants, which are resistant to ABA mediated inhibition of germination and/or post-germinative growth, several important components regulating ABA signaling in seeds and/or guard cells have been identified in *Arabidopsis thaliana* [2], [3], [7], [12]. Among them, ABI1 [13] and ABI2 [14] are protein phosphatases that negatively regulate ABA signaling during seed dormancy and germination. These phosphatases were also shown to be involved in ABA-mediated guard cell signaling as well [15]. In contrast, the ABI transcription factors including ABI3, ABI4 and ABI5 act positively to regulate ABA signaling in seeds [2], [16], [17], [18].

In plants, products of the lipoxygenases (LOXs) pathway have showed diverse functions involved in abiotic stress [19]. However, some results have suggested that LOXs play important roles in seed germination and seedling growth and development [20], [21]. Lipoxygenases are non-heme iron-containing dioxygenases widely distributed in plants and animals. LOX catalyzes the addition of molecular oxygen to polyunsaturated fatty acids containing a (*Z*,*Z*)-1,4-pentadiene system to produce an unsaturated fatty acid hydroperoxide, and initiates the synthesis of a group of acyclic or cyclic compounds collectively called oxylipins, which are products of fatty acid oxidation, with diverse functions in the cell. Feussner and Kindl reported that LOX is the main lipid body protein in cucumber cotyledons during the early stages of seed germination [20]. New LOXs are synthesized in the seedling and the cotyledon during germination. Maximal accumulation of LOXs proteins and the corresponding mRNAs lasts from a few hours to a few days after germination. However, the LOX mRNAs synthesized during germination also could be found in the mature plant, and their expression levels were increased by the application of abscisic acid [22]–[24].

Nordihydroguaiaretic acid (NDGA) is a phenolic compound that is present in high concentration in the leaves of the evergreen desert shrub Creosote bush, *Larrea tridentate*, which grows in some desert areas of southwest United States and northern Mexico [25]. Many studies have been shown that NDGA is a potent in vitro scavenger of peroxynitrite anion, singlet oxygen, hydroxyl radical, and hypochlorous acid [26]. The antioxidant properties of NDGA are attributed to its activity as a non-selective inhibitor of lipoxygenases, reducing the iron atom in the iron-enzyme complex to the inactive ferrous state [27]. The oxidative cleavage of certain xanthophylls that also occurs during ABA biosynthesis is mediated by a nonheme oxygenase with lipoxygenase-like properties [28], which furtherly was demonstrated that NDGA is an inhibitor of the NCED enzymes in maize [29]. Thus, NDGA is an ideal chemical to study the relationship between LOX pathway and ABA signaling in regulating the seed germination and post-germination growth.

The *CPR5* gene has recently been isolated and shown to encode a protein with 5 potential transmembrane regions at the carboxy terminus, a bipartite nuclear localization signal at the amino terminus, and no sequence similarity to other known proteins [30], [31]. *CPR5* appears to act just downstream of pathogen recognition and upstream of salicylic acid in a resistance pathway dependent on *NPR1* (*NONEXPRESSOR OF PATHOGENESIS RELATED GENES 1*) [32]. Boch and coworkers showed that *CPR5* activates the *PR* gene expression in the RPS2-mediated pathway [33]. However, CPR5 appears to play important roles in plant growth and development as well, because *cpr5* mutants exhibit defects in cell proliferation and expansion [30], and the gene also functions in cell wall biogenesis [34]. In addition, Yoshida and co-workers show that *cpr5* (*hys1*) mutants are hyperresponsive to glucose and sucrose and prematurely accumulate senescence upregulated transcripts [31]. All *cpr5* alleles isolated so far exhibit early cotyledon senescence, have areas of localized cell death on the rosette leaves, and have trichomes that are glassy and reduced in size and branching [30]–[33]. Thus, Jing and Dijkwel hence propose that *CPR5* is a master regulator of cellular ROS status and/or signaling [35], which has close and complex interactions with other signaling networks to control cell proliferation, endoreduplication and trichome development, responses to biotic and abiotic stress [35]. In this report, we provide new evidence that *CPR5* also plays important roles in the pathway controlling postgermination arrest of development through LOX pathway and ABA signaling pathway.

## Materials and Methods

### Plant Materials and Growth Conditions

The Arabidopsis thaliana ecotypes *Columbia*, *Landsberg erecta* were used throughout this study. The *cpr5* mutant allele used in this paper was *cpr5-1* [32]. Seeds were surface-sterilized for 2 min in 75% ethanol, followed by 5 min in 1% NaClO solution and washed five times in sterile distilled water, plated on growth medium (MS medium, 1.5% sucrose, 0.8% agar and pH 5.7). Plates were routinely kept for 2 days in the dark at 4°C to break dormancy (stratification) and transferred to a tissue culture room with a 16-h-light/8-h-dark cycle (light intensity of 120 mol m^−2^ s^−1^). After one week, seedlings were potted in soil and placed in a growth room at 22°C. The ABA-insensitive mutant *abi1-1* was used to generate double mutants with *cpr5-1*. The double mutant lines were created by cross-pollination between the relevant mutants, and putative double mutant plants were screened from the phenotype segregation ratio of the F2 progeny.

### Transformation Vectors and Construction of Transgenic Plants

Transgenic plants carrying constitutively expressing transgenes were generated. To produce *35S-CPR5* plants, a 1695 bp *Kpn*I-*Spe*I fragment containing the *CPR5* (The Arabidopsis Information Resource locus At5g64930) cDNA was cloned into the vector pCanG vector and verified by sequencing, in which transgene expression is under the control of the CaMV 35S promoter. For the *CPR5* promoter and GUS fusion constructure, a 741 bp promoter region just upstream of the ATG start codon of *CPR5* was amplified from genomic DNA by PCR. The PCR fragment was cloned into the *Sal*I -*BamH*I site of binary vector pBI101.1 to obtain a transcriptional fusion of the *CPR5* promoter and the GUS coding sequence. For the functional analysis of the transmembrane domains predicted in CPR5, a truncated form, CPR5ΔTM, with last transmembrane domains deleted (residues 525 to564) (ΔTM) was also cloned into the vector pCanG. To prepare the 35S-CPR5-GFP fusion construct, the entire coding region of *CPR5* was inserted directly upstream of the EGFP coding region in pBEGFP (pBEGFP is reconstructed based on pBin19). Plants were transformed with *Agrobacterium tumefaciens* by the floral dipping method [36]. Transgenic seeds were germinated on MS plates containing 50 mg/L kanamycin for pBI101.1, pCanG and pBEGFP, and the resistant plants were transferred to soil to obtain homozygous seeds. Two independent lines of homozygous plants containing a single insertion of each construct were used for detailed analysis.

### Histochemical Analysis and Confocal Microscopic Observation

The plants include the CPR5: GUS was assayed for the GUS color reactivity. The plant material was immersed in GUS staining solution (50 mM Na-Phosphate buffer, pH 7.0, 1 mM EDTA, 0.1% Triton X-100, 100 µg/ml chloramphenicol, 1 mg/ml X-Gluc, 2 mM Ferri cyanide, 2 mM Ferro cyanide) and incubated overnight at 37°C. The material was washed repeatedly in 95% ethanol until the tissue was bleached. The stained tissue was then observed and photographed using an Olympus BX51 Microscope (Olympus Corporation, Japan).

35S-CPR5-GFP plants were used for GFP subcellular localization analysis. Roots of 7-day-old transgenic seedlings were used for the green fluorescence analysis (GFP localization) by a Carl Zeiss laser scanning system LSM 510 (http://www.zeiss.com).

### ABA, NDGA Treatments and Seed Germination, Cotyledon Greening and Primary Root Length Measurements

Plants of different genotypes were grown in the same conditions and seeds were collected at the same time. For each comparison, seeds were planted in the same plate containing MS medium (0.5×MS salts, 1% sucrose, and 0.8% agar) without or with different concentrations of ABA, NDGA and tea polyphenols. Plates were chilled at 4°C in the dark for 2 d (stratified) and moved to 22°C with a 16-h light/8-h dark cycle. The percentage of seed germination was scored at indicated times. Germination was defined as an obvious emergence of the radicle through the seed coat. Cotyledon greening is defined as obvious cotyledon expansion and turning green. As primary root length measurements, plates were placed vertically in growth chamber. At indicated times, the plates were scanned by an Epson perfection V200 photo scanner, and the primary root length was measured by the tool DIGIMIZER 3.2.1.0 (http://www.digimizer.com).

### Drought Treatment and Measurement of Transpiration Rate

For the soil-grown plant drought tolerance test, one-week-old seedlings were transplanted to the soil for two weeks under standard growth conditions, and then plants were subjected to progressive drought by withholding water for specified times. To minimize experimental variations, the same numbers of plants were grown on the same tray. The entire test was repeated a minimum of three times. To measure the transpiration rate, detached fresh leaves were placed abaxial side up on open petri dishes and weighed at different time intervals at room temperature. Leaves of similar developmental stages (the third to the fifth rosette leaves) from 3-week-old soil-grown plants were used.

### Stomatal Aperture Measurements

Epidermal peels were stripped from fully expanded leaves of two week-old plants, and were floated in a solution of 30 mM KCl and 10 mM 2-(N-morpholine)-ethanesulphonic acid (MES-KOH; pH 6.15) in Petri dishes. ABA was added to the solution, and stomatal apertures were recorded under a light microscope (BX51; Olympus, http://www.olympus-global.com). Measurements were performed as described by Ichida et al [37] using the free software DIGIMIZER 3.2.1.0 (http://www.digimizer.com).

### Gene Expression Analysis

Real-time PCR was performed using the ABI Prism 7300 Fast Real-time PCR system (Applied Biosystems Inc.) with SYBR Premix Ex Taq (Takara Bio, Inc.). Total RNA was extracted as described above. cDNAs were synthesized from 0.5 µg of total RNA using PrimeScript™ RT reagent Kit (Perfect Real time) (Takara Bio, Inc.). Each PCR reaction contained 1× SYBR Premix Ex Taq, 0.2 µM of each primer, and 2 µl of a 1∶10 dilution of the cDNA in a final volume of 20 µl. The following PCR program was used: initial denaturation, 95°C, 15 s; 40 cycles of 95°C for 4 s, 60°C for15 s and 72°C for 31 s. In melting curve analysis, PCR reactions were denatured at 95°C, re-annealed at 60°C, then a monitored release of intercalator from PCR products or primer dimmers by an increase to 95°C. cDNA quantities were calculated by ABI Prism 7300 SDS Software Ver.1.3 (Applied Biosystems Inc.), and transcript data were normalized using *UBQ10* gene as an internal control. Relative expression levels of target genes in WT, *cpr5*, and *CPR5* over-expression plants with or without ABA treatments, were calculated based on corresponding levels in wild-type plants without ABA treatment. Error bars were presented to indicate the standard error of the mean. All experiments were performed with three replicates. The primers used in this paper for Quantitative RT-PCR are listed in Supplemental Table S1.

## Results

### Expression Pattern of *CPR5*

**Figure 1:**
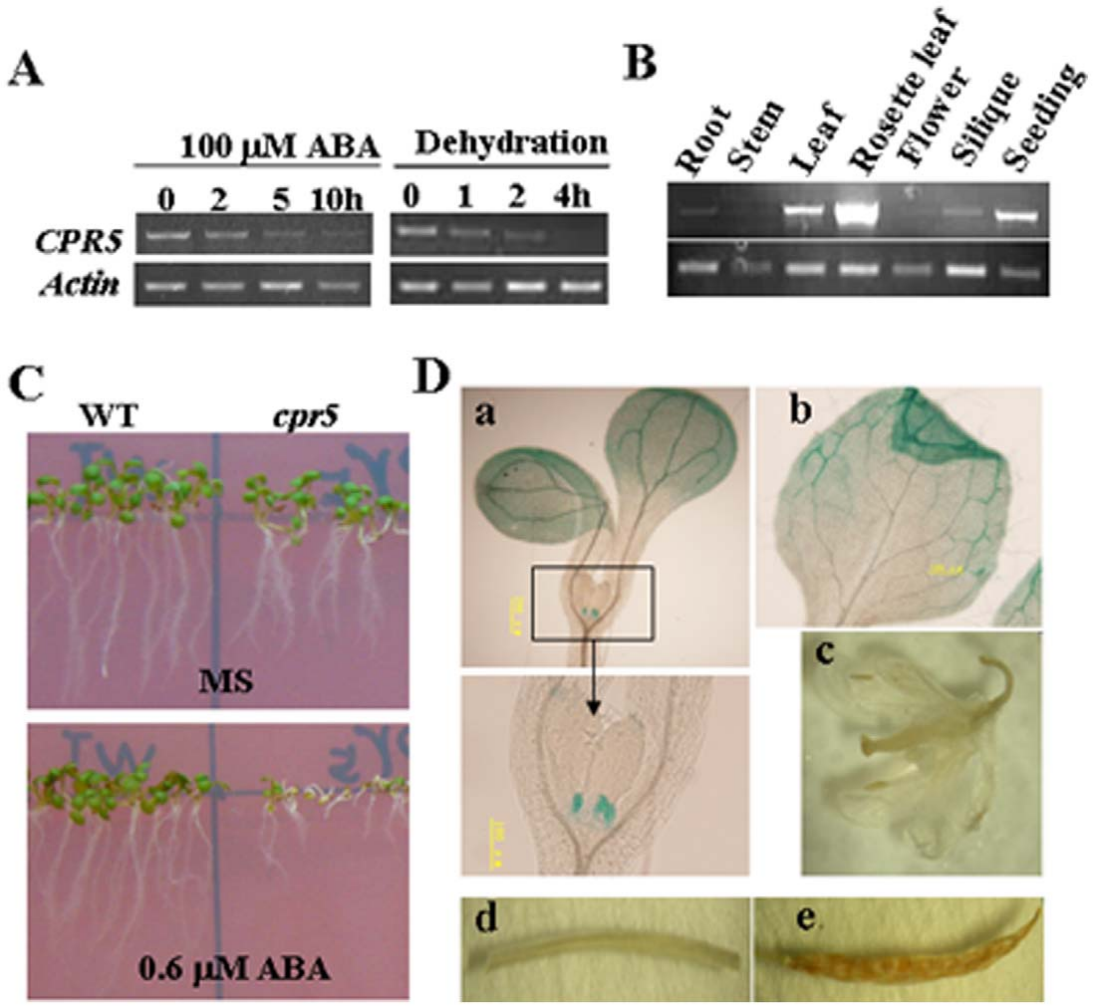
Expression patterns of *CPR5*. A. Expression patterns of *CPR5* gene transcripts in response to 100 µM ABA and dehydration treatment. Numbers indicate the time of treatment (hours) B. RT-PCR analysis of *CPR5* transcripts in different tissues of Arabidopsis plants. Total RNA was isolated from various tissues (root, leaf, stem, flower and silique) of wild-type plants grown under long-day conditions. C. Growth phenotype of *cpr5* and wild type plants on MS medium containing 0 µMABA and 0.6 µMABA. D. Histochemical (β-glucuronidase) GUS analysis of *CPR5* promoter-GUS transgenic plant. (a) four-day old seedling; (b) rosette leaf; (c) flower; (d) stem; (e) silique.

To gain further insights into possible functions of *CPR5*, the expression pattern of this gene was examined. RT-PCR analysis detected *CPR5* in all tissues of *Arabidopsis*, including root, leaf, siliques and seeding (Figure 1B). To determine the expression pattern in detail, the expression of the *CPR5* promoter–β-glucuronidase (GUS) fusion was analyzed. Histochemical staining revealed GUS activity mainly at the cotyledons in seedlings (Figure 1D). Interestingly, strong GUS staining was specifically observed in the true leaf emergence site (Figure 1D).

To investigate whether *CPR5* is involved in abiotic stress, two-weeks-old *Arabidopsis* seedlings were subjected to different stress treatments and RNA was extracted for gene expression analysis. Semi-quantitative RT-PCR showed that *CPR5* transcript accumulation was decreased after exogenous application of 100 µM ABA or dehydration stress (Figure 1A). To elucidate the possible role of *CPR5* in response to ABA, *cpr5-1* and wide type seeds were germinated on MS medium with or without ABA, the growth of *cpr5-1* plants was arrested compared with that of the wild type (Figure 1C). This indicated an enhanced susceptibility of *cpr5-1* plants to ABA.

### ABA Response of *cpr5-1* and *35S-CPR5* Plants

**Figure 2:**
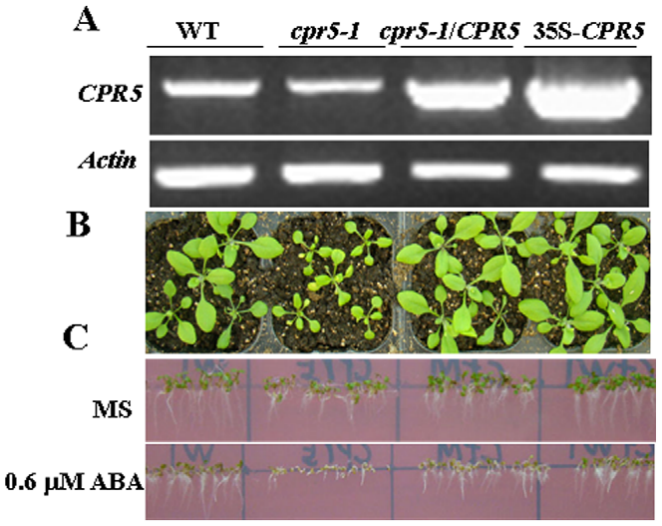
Growth phenotypes of *cpr5-1* and *CPR5* transgenic plants. A. *CPR5* transcripts in wild-type, *cpr5-1* and transgenic lines *cpr5*/*CPR5* and *35S-CPR5*. B. Phenotypes of wild type, *cpr5-1*, *cpr5-1/CPR5* and *35S-CPR5* plants grown on soil for15 days. C. Growth phenotype of transgenic and mutant plants on MS medium containing 0 µM ABA and 0.6 µM ABA. Seeds were germinated and grown for 7 days.

ABA plays an important role in regulating plant responses to different stresses [2]. Inhibitory experiments of seed germination have provided useful insights into components of ABA signaling [38]. Since *CPR5* is an ABA repressed gene and *cpr5-1* was sensitive to exogenous ABA (Figure 1A, C), it is possible that *CPR5* plays a role in plant responses to ABA. To confirm whether the phenotypes of the *cpr5-1* mutants above were indeed due to the *CPR5* function loss, the *CPR5* cDNA under the control of the cauliflower mosaic virus (CaMV) 35S promoter was used to transform the *cpr5-1* mutant and wild type plants. The complementation and overexpression lines had clearly increased expression of *CPR5* confirmed by RT-PCR (Figure 2A); In addition, the leaf mimic and ABA hypersensitivity were rescued in the complementation lines compared to that of the *cpr5-1* mutant (Figure 2B, C). These results indicated that the ABA hypersensitivity of *cpr5-1* mutant was due to the *CPR5* function loss. Thus, *cpr5-1* mutant and *CPR5* overexpression lines were used for all further studies.

**Figure 3:**
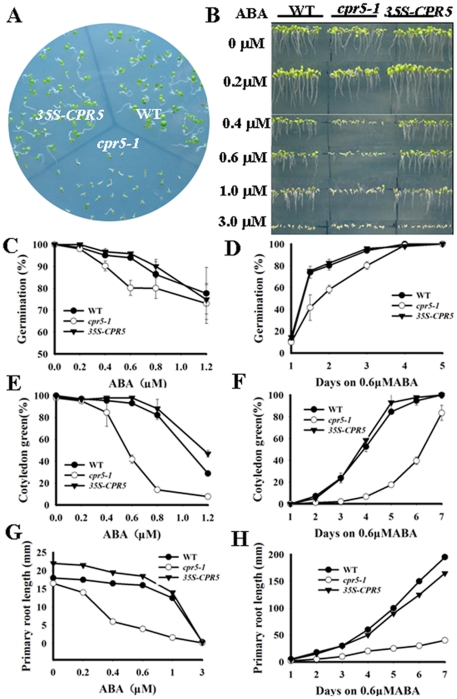
Responses to ABA of *cpr5-1* and *35S-CPR5* plants. A. Growth of different genotypes of plants on MS medium containing 0.6 µM ABA. Seeds were germinated for 6 days after stratification. B. Primary root growth of wild-type, *cpr5-1* and *35S-CPR5* seedlings on MS medium containing a range of concentrations (0 µM, 0.2 µM, 0.4 µM, 0.6 µM, 1.0 µM and 3.0 µM) of ABA. Seeds were germinated for 7 days after stratification. C. Quantification of seed germination. Seed germination percentage of three genotypes grown on different concentrations of ABA was recorded at 3 days after stratification. D. Seed germination time course of three genotypes grown on medium containing 0.6 µM ABA. E. Quantification of cotyledon greening. Cotyledon-greening percentage of three genotypes grown on medium containing different concentrations of ABA was recorded at 6 days after stratification. F. Cotyledon greening time course of three genotypes grown on medium containing 0.6 µM ABA. G. Root growth measurements. Seedling root length of three genotypes grown on different concentrations of ABA was measured at 7 days after stratification. H. Primary root growth time course of three genotypes grown on medium containing 0.6 µM ABA. For A to H, at least three independent experiments were conducted and similar results were obtained.

To determine the role of *CPR5* in ABA signaling, the seeds of wild-type, *cpr5-1* mutant and *CPR5* overexpression lines were germinated on MS medium containing 0 µ M, 0.2 µM, 0.4 µM, 0.6 µM, 0.8 µM ABA, 1.0 µM, 1.2 µM ABA, and compared for the differences in germination and postgerminative growth. In the absence of ABA, there was no significant difference among wild-type, *cpr5-1* and *CPR5* overexpression lines. In the presence of ABA, the ABA-sensitive response of *cpr5-1* occurred at concentrations as low as 0.4 µM ABA, *cpr5-1* germinated later than wild type (Figure 3C, D). In addition, the *cpr5-1* mutant and *CPR5* overexpression plants were also assessed for their response to ABA during early seedling development. The cotyledon greening of *cpr5-1* was severely inhibited on MS medium with 0.6 µM ABA (Figure 3A) , 98% of *35S-CPR5*, 95% of wild-type but only 40% of *cpr5* seedlings had green cotyledons after germinated for 6 days (Figure 3F). When ABA concentration reached to 1.2 µM, the growth of *cpr5-1* mutant seedlings was severely arrested and few of them developed true leaves at 8 days (Figure 3E). Furthermore, the action of *CPR5* in ABA signaling was also assessed by investigating the ABA-mediated retardation of seedling root growth. In an ABA dose-response assay, seeds were germinated and growth on MS medium containing 0 µM, 0.2 µM, 0.4 µM, 0.6 µM, 1.0 µM and 3.0 µ M ABA. The root growth of the *cpr5* plants was more severely inhibited in the presence of different concentrations of ABA (Figure 3B). The root growth rate of the *cpr5-1* seedlings was less than that of the wild-type plants and the sensitivity could be reversed by *CPR5* overexpression (Figure 3H). At all concentrations tested, the *cpr5-1* mutant was hypersensitive to ABA at both germination and postgerminative growth stages, and the sensitivity occurred in a dosage-dependent manner (Figure 3C, E, G). Taken together, the contrasting ABA sensitivities displayed by the *cpr5-1* mutant and *CPR5* overexpression plants suggested that *CPR5* play important roles in ABA signaling during germination and early seedling development.

**Figure 4:**
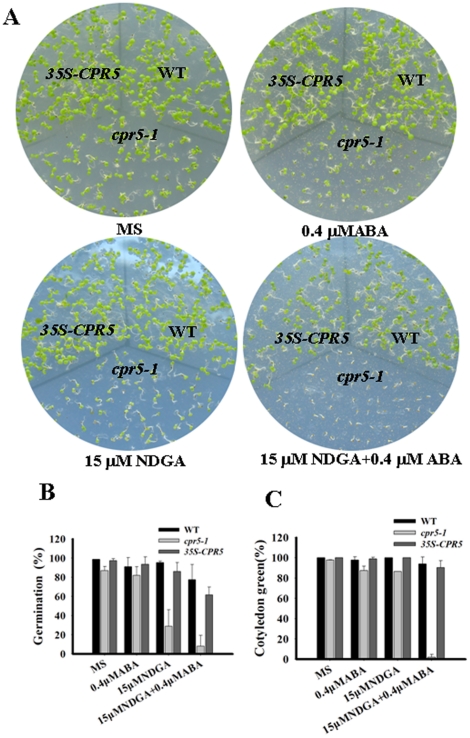
The influence of NDGA on ABA sensitivity of *cpr5-1* and *35S-CPR5* plants. A. Growth of different genotypes plants on MS and MS medium containing 0.4 µM ABA, 15 µM NDGA and 15 µM NDGA+0.4 µM ABA. Seeds were germinated and grown for 7 days. B. Germination percentage of three genotypes plants on different mediums discribed in a. Germination was scored at 2 days after stratification. C. Cotyledon greening percentage of three genotypes plants on different mediums discribed in A. Cotyledon greening was scored at 7 day after stratification.

It has been shown that fluridone, an inhibitor ABA biosynthesis, can effectively prevent prevent the biosynthesis of ABA and reduced the ABA sensitivity of different genotypes [11], [39]–[41]. Nordihydroguaiaretic acid (NDGA) also acts as an inhibitor of the NCED enzymes regard to its permeation speed and ability to block ABA biosynthesis [29]. To challenge the possibility that *CPR5* may regulate ABA response through ABA biosynthesis, we examined the influence of NDGA and fluridone on ABA sensitivity of *cpr5-1* and *35S-CPR5* plants. Given that NDGA and fluridone are inhibitors of the ABA biosynthesis, we reasoned that if *CPR5* regulated ABA response through ABA biosynthesis, the inhibition of exogenous ABA should be partly alleviated by NDGA or fluridone treatment. When seeds were pretreated with 100 mM of fluridone, no difference was observed in the inhibition of exogenous ABA on seed germination and the cotyledon greening (Figure S1). Interestingly, it was found that NDGA treatment aggravated the ABA-induced delay in the seed germination and cotyledon greening assays (Figure 4). After 7 days, the cotyledon green rate of *cpr5-1* seedlings is 86 and 87% on MS medium with 0.4 µM ABA and 15 µM NDGA respectively, but only 2% of *cpr5* could germinate and develop to green seedlings when grown on MS medium with both 15 µM NDGA and 0.4 µM ABA (Figure 4C). It was found that, similar to the situation of cotyledon greening assay, *cpr5* displayed much stronger ABA and NDGA hypersensitivity than single ABA or NDGA in the ABA inhibition of the seed germination assay (Figure 4B). These data suggested that *CPR5* affects ABA signaling rather than ABA biosynthesis, and also might involved in NDGA-mediated seed germination and postgermination arrest of development.

### Drought Response of *cpr5* and *35S-CPR5* Plants

**Figure 5:**
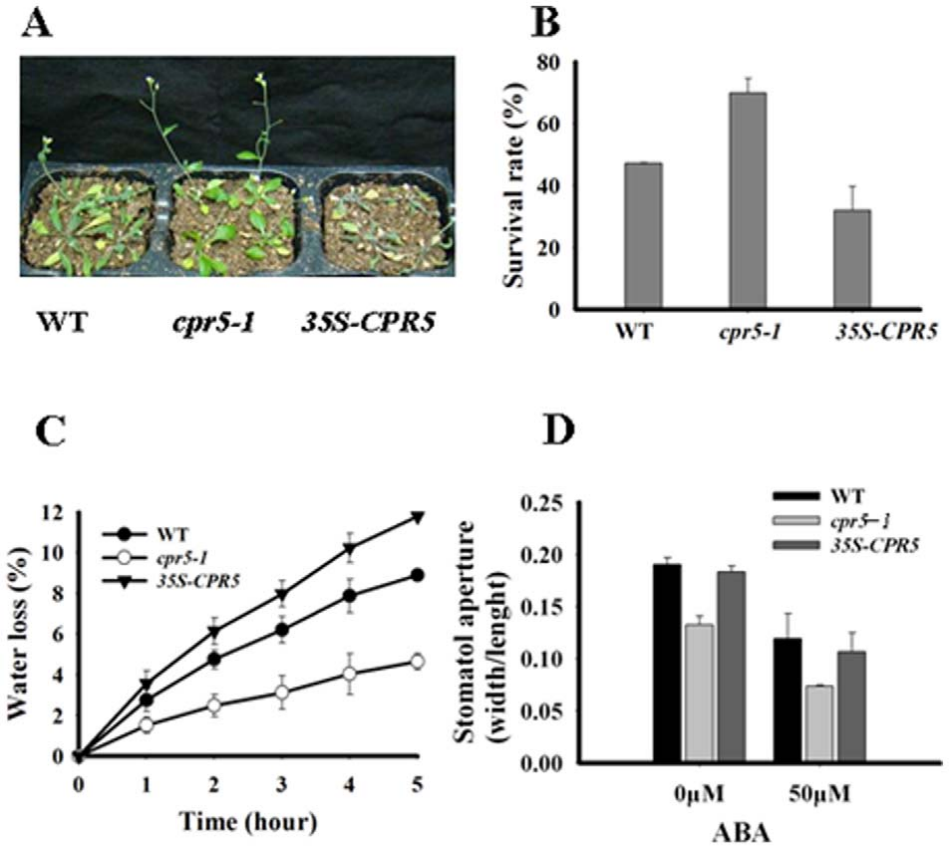
Responses to drought of *cpr5-1* and *35S-CPR5* plants. A. Drought tolerance of different genetype plants were grown on soil in the same container for 2 weeks, withheld from water for 18 days, and then rewatered for 2 days. B. Comparison of plants survival rates under the conditions described in A. C. Transpiration rates. Water loss during a 5-h period was measured using detached leaves from 3-weeks-old plants. D. Effects of ABA on stomatal aperture in wild-type, *35S-CPR5*, and *cpr5-1* plants. Data are mean ratios of width to length.

Because the *CPR5* gene was down-regulated by dehydration, it is expected that *cpr5-1* mutant may have altered response to water deficition. To test this, one-week old wild type, *cpr5-1* and *35S-CPR5* plants were transplanted to grow on soil for an additional two weeks. Thereafter, plants were challenged with drought by withholding water for 10 days. The plants were then rewatered and photographed after two days (Figure 5A), and the survived plants were measured. The *cpr5-1* mutant exhibited a high survival rate (76%), whereas the corresponding survival rate was 47% for wild type, and 27% for the *35S-CPR5* plants (Figure 5B). The altered drought tolerance of *cpr5* mutant plants could be attributed, at least in part, to changes in transpiration rate. To test this, leaf water loss of the wild type, *cpr5-1* and *35S-CPR5* plants was compared. After detached for 5 hours, the leaf water loss in *cpr5-1* plants was less than 5%, as opposed to 9% and 12% for wild type and *35S-CPR5* transgenic plants, respectively (Figure 5C). These results suggested that *CPR5* plays an important role in plant drought response.

The rate of plant water loss during drought is largely determined by stomatal aperture/closure, and stomatal closure is a key ABA-controlled process that determines the rate of transpiration under water deficit conditions [6]. To investigate whether *CPR5* is involved in ABA-related stomatal closure, we treated leaves of three genotypes with ABA to analyze stomatal aperture. Interestingly, stomatal aperture of *cpr5-1* was the smallest both in light-induced stomatal opening and ABA inhibition of light-induced stomatal opening (Figure 5D). However, no differences were observed in three genotypes in ABA inhibition assay of light-induced stomatal opening. The results suggested that *CPR5* may play a crucial role in guard cell control, but may be an ABA independent pathway.

### Expression of Stress-Responsive Genes in *cpr5-1* and *35S-CPR5* plants

**Figure 6:**
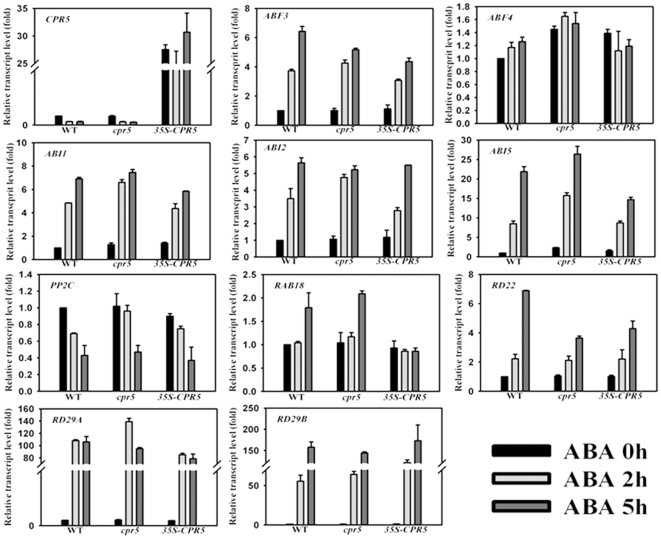
Expression of ABA and stress-responsive genes in *cpr5-1* and *35S-CPR5* plants. Ten-day-old seedlings grown on MS medium were treated with ABA (100 µM) for different time periods , expression of ABA- and stress-responsive genes with specific primers were analyzed by Real-time PCR, *UBQ10 gene* was used as an internal control.

The enhanced tolerance of *cpr5-1* plants to drought, along with the *CPR5* involved in ABA signaling during germination and postgermination growth and the inhibitory effect of ABA on *CPR5* gene expression in wild type plants, prompted us to evaluate whether the expression of ABA-responsive genes in *cpr5-1* mutant plants were affected. In order to address this question, the expression pattern of some stress marker genes was monitored. This analysis includes the widely researched *ABI1*, *ABI2*, *ABI5*, *AtPP2C*, *ABF3*, *ABF4*, *RD29A*, *RD29B* and *RD22* genes. As shown in Figure 6, ABA treatment induced the expression changes of these marker genes in varying degrees, and no much difference were observed in these stress marker genes except *ABI1* and *RAB18* among the wild type, *cpr5-1* and *35S-CPR5* plants. This indicated that the enhanced susceptibility to ABA exhibited by *cpr5* plants was at least partially independent of the above mentioned marker genes.

**Figure 7:**
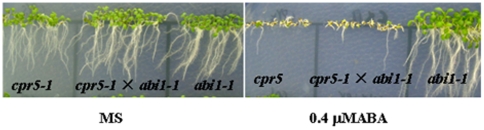
Double mutant analysis between *cpr5-1* and *abi1-1*. Seeds of different genotypes were germinated and grown vertically on MS containing 0 µM ABA and 0.4 µM ABA, the pictures were taken at 10 days after stratification.

The *cpr5-1* plants are hypersensitive to ABA; this phenotype is opposite to *abi1-1* mutant which is a dominant-negative type mutation. To test the genetic relationship between *CPR5* and *ABI1*, double mutant *cpr5-1 abi1-1* was generated by crossing *cpr5* with *abi1-1*, and its response to ABA was compared with that of the two single mutants. Given that *cpr5-1* is sensitive to ABA while *abi1-1* is insensitive to ABA, we reasoned that if *CPR5* act in the same pathway to *ABI1*, the double mutant should be exhibited either the *cpr5-1* or the *abi1* sensitivity to ABA. In our ABA response assays, double mutant *cpr5-1 abi1-1* plants were also sensitive to ABA (Figure 7). These results suggested that *CPR5* gene may be located downstream of the *ABI1* in the ABA signaling pathway.

### NDGA and Tea Polyphenols Response of *cpr5* and *35S-CPR5* Plants

**Figure 8:**
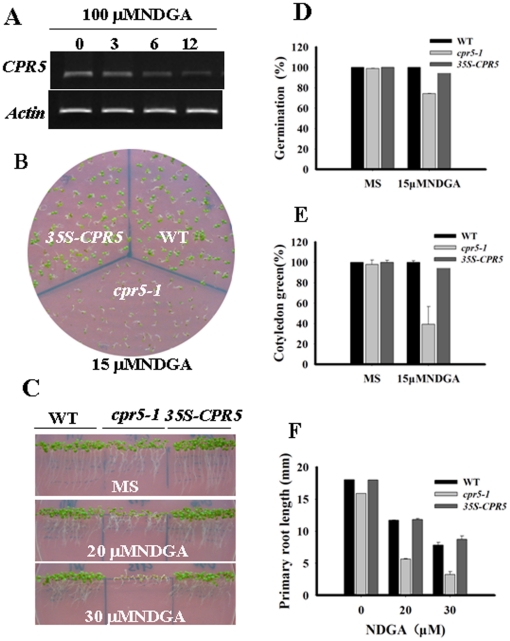
Responses to NDGA of *cpr5-1* and *35S-CPR5* plants. A. Expression patterns of *CPR5* gene transcripts in response to 100 µM NDGA. Numbers indicate the period of treatment (hours). B. Growth of different genotypes plants on MS medium containing 0 µM NDGA and 15 µM NDGA. Seeds were germinated and grown for 7 days. C. Primary root growth of different genotypes plants grown vertically on MS medium containing a range of concentrations (0 µM, 20 µM, and 30 µM) of NDGA. Seeds were germinated and grown for 8 days after stratification. D Quantification of seed germination. Seed germination was recorded at 3 days after stratification. E Quantification of cotyledon-greening. Cotyledon-greening was recorded at 7 days after stratification. F Root growth measurements. Seedling root length was measured at 8 days after stratification. All data show the mean ±SD of three experiment replicates.

**Figure 9:**
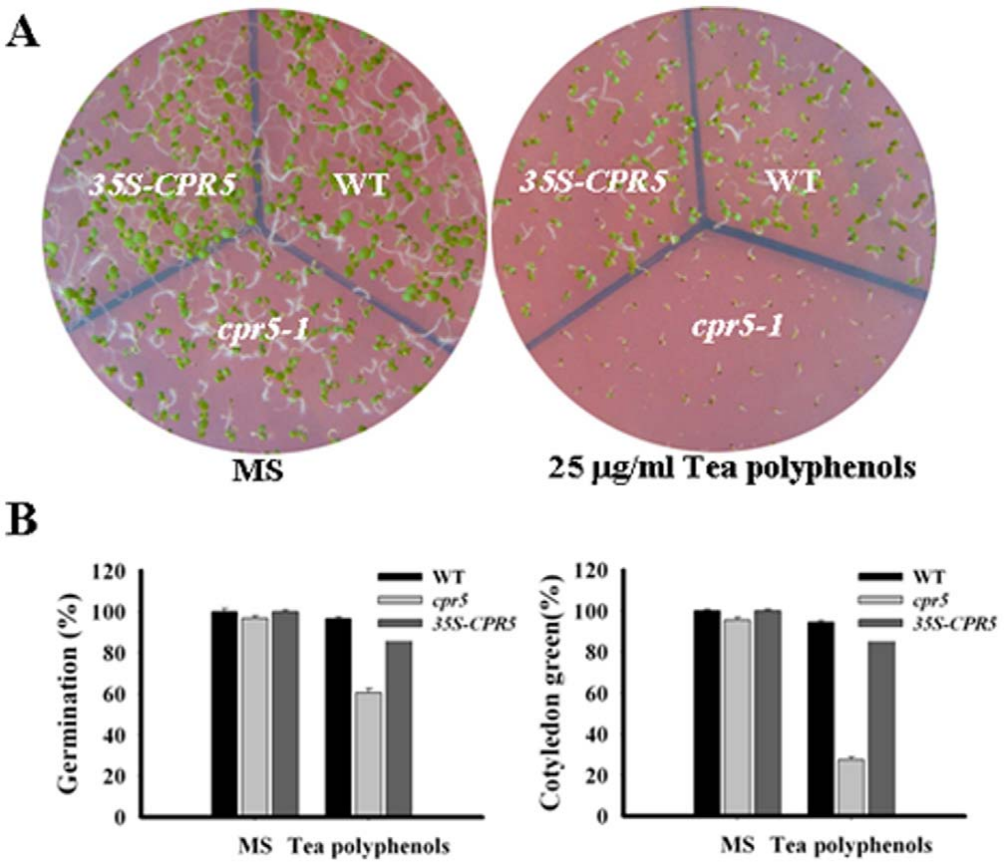
Responses to tea polyphenols of *cpr5-1* and *35S-CPR5* plants. (a) Growth of different genotypes plants on MS medium containing 0 µg/ml and 2 µg/ml tea polyphenols. Seed were germinated and grown for 7 days. (b) Germination and cotyledon greening percentage of different genotypes plants on the mediums discribed in A. Germination and cotyledon greening was scored at 3 days and 7 days after stratification.

Because *cpr5* was found to be NDGA-hypersensitive, and NDGA is a general inhibitor of the LOX activity, it is possible that *CPR5* may mediate the LOX pathway. As in NDGA response assays, *CPR5* was down regulated by exogenous NDGA (Figure 8A). In an NDGA dose-response assay, seeds were germinated and growth on MS medium containing 0 µM, 20 µM and 30 µM NDGA. The germination of the *cpr5-1* seed was slightly delayed; at 3 days, about 80% of the *cpr5-1*, but nearly100% of the wild type and *35S-CPR5* seeds germinated on medium containing15 µM NDGA (Figure 8d). In fact, the cotyledon greening and expansion (Figure 8B), as well as the root growth of the *cpr5-1* mutant (Figure 8C, F), were also inhibited at the postgermination stage, and at 7 days, only 40% of the *cpr5* plant had small green cotyledons (Figure 8E). These results indicated that *CPR5* is involved in the LOX pathway. In order to further confirm that, it was necessary to examine whether *cpr5* mutants were also sensitive to other inhibitors of the LOX pathway. As expected, *cpr5-1* mutants were also hypersensitive to tea polyphenols (another inhibitor of the LOX pathway) observed in germination assay scored by radicle emergence as well as in assays measuring the emergence of green cotyledons (Figure 9). These results demonstrated that *CPR5* mediated the LOX pathway during seed germination and postgermination arrest of development.

### Functional importance of the Transmembrane Domains of CPR5

**Figure 10:**
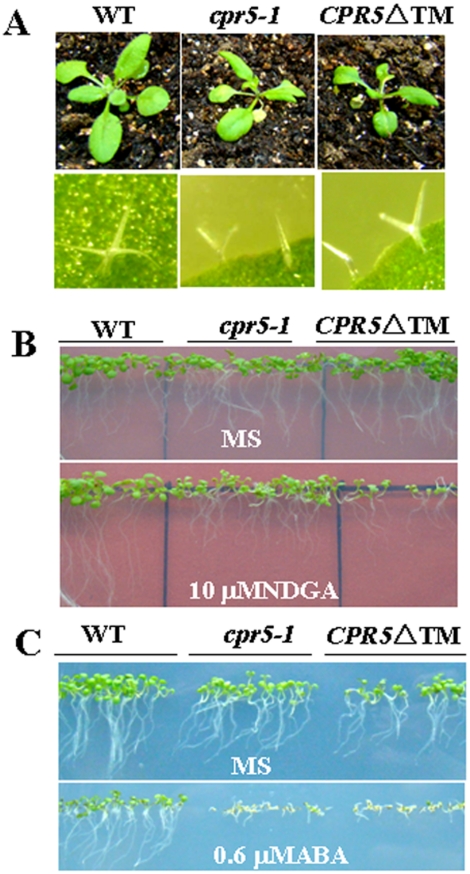
Functional importance of the CPR5 transmembrane domains. A. Aerial parts phenotype (top panel) and their leaf trichomes (bottom panel) of representative seedlings. B. Growth of different genotypes plants on MS containing 0 µM NDGA and 10 µM NDGA. Seeds were germinated and grown for 10 days. C. Growth of different genotypes plants on MS containing 0 µM ABA and 0.6 µM ABA. Seeds were germinated and grown for 8 days.

*CPR5* was predicted to encode a protein containing an amino terminus bipartite nuclear localization signal and five transmembrane domains at the carboxy terminus. To determine whether transmembrane domains were indispensable to its function, the CPR5 protein with a truncated form, CPR5ΔTM, with the last transmembrane domains deleted were constructed and expressed in *cpr5* mutant. As shown in Figure 10, the transformants CPR5ΔTM also exhibited *cpr5-1* phenotype. For example, at maturity, the transformants were much smaller than the wild type and their leaf trichomes were reduced in size and branching (Figure 10A). Furthermore, in root growth assays, the transformants were also hypersensitive to ABA and NDGA as compared with the wild type (Figure 10B, C). All together, the results of our deletion analysis show that the transmembrane domains of CPR5 are important to maintain the function and its capacity to respond to ABA and NDGA.

### The CPR5 protein is localized to the Cytoplasm

**Figure 11:**
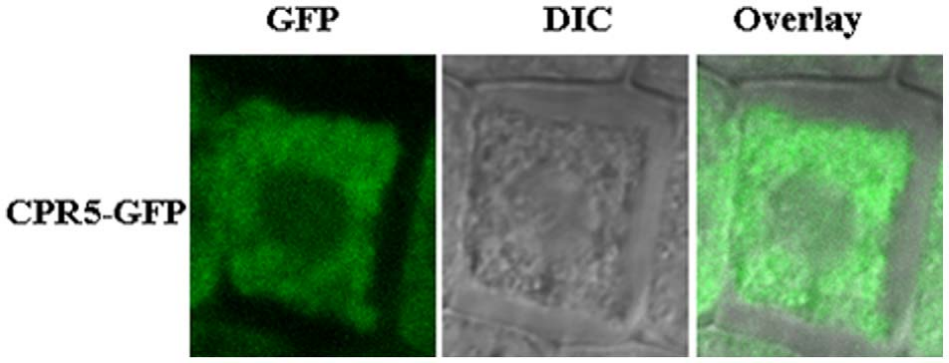
Subcellular localization of the CPR5. CPR5-GFP in arabidopsis root cells were analysed by confocal microscopy.

So far, more *cpr5* alleles were identified and pleiotropy functions were found, however, the subcellular localization of the CPR5 protein remains unknown. To verify its sub-cellular localization, CPR5 was fused (C-terminal) with EGFP and permanently expressed in *cpr5-1* mutant under the control of the CaMV 35S promoter. The 35S-CPR5-EGFP fusion transgenic plant can complement the phenotype of *cpr5-1* mutant (Figure S3), demonstrating that CPR5 fused to EGFP functioned similarly to CPR5 alone. The cytoplasm signal was visualized in 35S-CPR5-EGFP complement root cells by confocal microscopy (Figure 11). Thus, our results demonstrated a cytoplasmic localisation of the CPR5 protein in this assay.

## Discussion

*CPR5* encode a protein with five potential transmembrane regions at the carboxy terminus which is involved in several processes including cell death, cell cycle, pathogen response, dark-induced leaf senescence, and also severely affected in the ploidy levels of trichomes and exhibit a marked reduction in cell number [30], [31]. In this study we provide evidence that *CPR5* controls a drought response. We propose that *CPR5* is an early negative regulator of ABA signal transduction related to this process. In addition, we also found that *CPR5* negatively regulates germination and early seedling growth through LOX pathway. Our results also suggested that *CPR5* independently regulates seed germination and postgermination arrest of development through LOX pathway and ABA signaling.

### CPR5 is a Cytoplasm Localization protein with Transmembrane domain

CPR5 is predicted to be a Type IIIa membrane protein with five transmembrane helices at the C terminus and a cytoplasmatic N terminus with a putative nuclear localization sequence(NLS), it has also been proposed that the protein may be involved in a signaling cascade in which the cytoplasmic domain is proteolytically cleaved and transported into the nucleus [30], However, our results demonstrated that CPR5 is a cytoplasmic protein not nuclear localization protein in Arabidopsis suggesting that the predicted NLS in the cytoplasmatic N terminus maybe not a real functional NLS (Figure 11). Furthermore, our results had also demonstrated that its transmembrane domains are important to maintain its function (Figure 10). *CPR5* is involved in several processes including pathogen responses, cell proliferation, cell expansion, dark-induced leaf senescence, and also severely affected in the ploidy levels of trichomes and exhibit a marked reduction in cell number, a possible model to explain the extreme pleiotropy of the *cpr5* mutant phenotype that is the CPR5 protein may be required directly for the function of multiple transcription factors involved in a wide range of distinct processes [34]. In fact, some recent work demonstrated that some transcription factors are localized to the nucleus and cytoplasm, which interact with cytosolic protein [42]. Thus, we proposed that CPR5 protein may interact with multiple cytosolic-nucleus localization proteins including transcriptional factor or other cytosolic proteins for its pleiotropy functions.

### *CPR5* is a negative Regulator of the ABA-mediated Seed Germination, Postgermination Growth and Drought Response

ABA is an essential mediator in triggering plant responses to most of the common abiotic stresses, including drought, salinity, high temperature, oxidative stress, and cold [2]. Nevertheless, high levels of ABA inhibit plant growth by affecting cell division and elongation [2]. Our data suggest that increase in endogenous ABA (provoked by dehydration treatment), as well as the exogenous application of ABA, resulted in decreased levels of *CPR5* transcript (Fig. 1. A). The loss of function of *CPR5* resulted in increased sensitivity to ABA, as demonstrated by comparison of the ABA-mediated plant growth inhibition in *cpr5-1* and wild type plants (Figure 3).

On the other hand, *cpr5* mutant plants showed an increased tolerance to drought (Figure 5A,B). Measurements of water loss in detached leaves from irrigated plants showed faster water loss in *cpr5-1* mutant (Figure 5C). The ability of guard cells to respond to environmental changes and close when water available, is one of the major mechanisms that govern water loss in plants [43], [44]. Since ABA regulates stomatal activity [45], the stomatal response to ABA was also examined in three genotypes. Interestingly, stomatal aperture of *cpr5-1* was the smallest both in light-induced stomatal opening and ABA inhibition of light-induced stomatal opening (Figure 5D). However, these changes were of a similar magnitude in three genotypes in ABA inhibition assay of light-induced stomatal opening. The results suggested that *CPR5* may play a crucial role in drought tolerance response by regulating stomatal opening, but may be an ABA independent pathway.

In addition, our findings that mutation or overexpression of *CPR5* did not significantly affect ABA-induced marker gene expression support that the ABA sensitivity and drought tolerance exhibited by *cpr5-1* plants was not conferred by an elevated or faster induction of the expression of these marker genes except *ABI1* (Figure 6), which is similar with the results of Bu *et al* (2009) [46] and Ramirez et al (2009) [47] who found *rha2a* and *ocp3* mutants are hypersensitive to ABA, while ABA induced marker genes were unaltered in both mutants.

Furthermore, molecular genetics studies have significantly advanced our understanding on the molecular basis of ABA signaling in seeds and seedlings. The *cpr5-1* plants are hypersensitive to ABA; this phenotype is opposite to *abi1-1* mutant which is a dominant-negative type mutation. To test the genetic relationship between *CPR5* and *ABI1*, double mutant *cpr5-1 abi1-1* was generated by crossing *cpr5-1* with *abi1-1*, and its response to ABA was compared with that of the two single mutants. Given that *cpr5-1* is sensitive to ABA while *abi1-1* is insensitive to ABA, we reasoned that if *CPR5* act in the same pathway to *ABI1*, the double mutant should be exhibited either the *cpr5-1* or the *abi1* sensitivity to ABA. In our ABA response assays, double mutant *cpr5-1 abi1-1* plants were also sensitive to ABA (Figure 7). Taken together, these results support the view that *CPR5* gene may be located downstream of the *ABI1* in the ABA signaling pathway.

All these results demonstrated the conclusion that *CPR5* is involved in ABA mediated seeds germination, postgermination growth and drought tolerance.

### *CPR5* independently Regulates Seed Germination and Postgermination Growth through LOX Pathway and ABA Signaling

NDGA is an ideal inhibitor of lipoxygenase and is attributed to its activity as a non-selective inhibitor of lipoxygenases ([48], however, Ren *et al* [49], [29] reported that Nordihydroguaiaretic acid (NDGA) is an ideal inhibitor of the NCED enzymes, 100 µM NDGA could totally blocked ABA accumulation. and was used to study the relationship of glucose-induced delay of seed germination in rice and ABA biosynthesis [50]. Lipoxygenases (LOXs, linoleate:oxygen oxidoreductase; EC 1.13.11.12) catalyze the regio-and stereo-specific oxygenation of polyunsaturated fatty acids to their corresponding hydroperoxy derivatives [51]. Oxidation of fatty acid is essential if lipids are to be used as a carbon source during early stages of seed germination [52]. Increases in lipoxygenase activity during germination have been reported for a number of plant species, including soybean [53], barley [54], rice [55], cucumber [56] and *Brassica napobrassica* [21].

It has been shown that fluridone, an inhibitor ABA biosynthesis, can effectively reduce endogenous ABA levels. Pretreatment with fluridone generally reduced the ABA sensitivity of different genotypes [11], [39]–[41]. Given that NDGA is also an inhibitor of the ABA biosynthesis, the inhibition of exogenous ABA should be partly alleviated by NDGA treatment. However, this study found that NDGA treatment aggravated the ABA-induced delay in the seed germination and cotyledon greening assays (Figure 7). The finding that NDGA treatment did not diminish the difference in ABA-mediated inhibition of seed germination and cotyledon greening among genotypes suggested that *CPR5* does not involved in ABA biosynthesis. In the other hand, NDGA is also an ideal inhibitor of lipoxygenase, the NDGA and tea polyphenols sensitivity of *cpr5-1* mutant (Figure 8 and Figure 9) indicates that *CPR5* may have a role in LOX pathways responses.

The formation of oxylipins starts with the conversion of polyunsaturated fatty acids (PUFAs). Initial conversion of PUFAs by lipoxygenases (LOXs) [57] or by a-dioxygenase (a-DOX) [58]. Subsequent conversion of hydroperoxides can occur by various alternative pathways, including those initiated by allene oxide synthase (AOS), divinyl ether synthase (DES), hydroperoxide lyases (HPL), peroxygenases (POX), or epoxy alcohol synthase (EAS). The resulting oxygenated derivatives include the phytohormone JA, as well as oxylipins with characteristic reactive epoxide, a, b-unsaturated carbonyl, or aldehyde functionalities. Jasmonic acid (JA) and some of its precursors and derivatives are signal molecules that function as essential mediators of the plant's wound, anti-herbivore and anti-pathogen responses, as well as in growth and development [38], [59], [60]. In our MeJA response assays, no obvious differences were observed in wild type, *cpr5-1* and *35S-CPR5* transgenic lines (Figure S2), which suggested that the NDGA sensitivity *cpr5* displayed may be independent the AOS branch in the LOX pathway.

In summary, our observations indicate that *CPR5* plays a regulatory role in regulation of seed germination and early seedling growth through the ABA and LOX pathways, both pathways appear to work independently, but are both regulated by *CPR5*.

## Supporting Information

Figure S1 **The influence of fluridone on ABA sensitivity of** ***cpr5-1*** **and** ***35S-CPR5*** **plants.** Matched seed lots were pretreated with deionized water or 100 mM fluridone for 24 h at 4°C before being placed at 22°C for germination. Seeds were germinated on MS and MS medium containing 0.4 µM ABA, and grown for 5 days.
(TIF)

Figure S2 **MeJA response analysis of** ***cpr5-1*** **and** ***35S-CPR5*** **plants.** Seeds of wild type, *cpr5*, *35S-CPR5* plants were germinated and growth for 12 days on MS medium containing 0 µM and 50 µM MeJA.
(TIF)

Figure S3 **35S-CPR5-EGFP fusion transgenic plant complements the phenotype of** ***cpr5-1*** **mutant.** Two-week-old seedlings of 35S-CPR5-EGFP in *cpr5-1* showing the phenotype of the wild-type.
(TIF)

Table S1 **Primers used in this study.**
(DOC)

## References

[1] Lopez-Molina L, Mongrand S, Chua NH (2001). A postgermination developmental arrest checkpoint is mediated by abscisic acid and requires the *ABI5* transcription factor in Arabidopsis.. Proc Natl Acad Sci U S A.

[2] Finkelstein RR, Gampala SS, Rock CD (2002). Abscisic acid signaling in seeds and seedlings.. Plant Cell.

[3] Nambara E, Marion-Poll A (2003). ABA action and interactions in seeds.. Trends in Plant Science.

[4] Himmelbach A, Yang Y, Grill E (2003). Relay and control of abscisic acid signaling.. Curr Opin Plant Biol.

[5] Chow B, McCourt P (2004). Hormone signalling from a developmental context.. J Exp Bot.

[6] Leung J, Giraudat J (1998). Abscisic Acid Signal Transduction.. Annu Rev Plant Physiol Plant Mol Biol.

[7] Assmann SM (2003). OPEN STOMATA1 opens the door to ABA signaling in Arabidopsis guard cells.. Trends in Plant Science.

[8] Christmann A, Moes D, Himmelbach A, Yang Y, Tang Y (2006). Integration of abscisic acid signalling into plant responses.. Plant Biol (Stuttg).

[9] Yamaguchi-Shinozaki K, Shinozaki K (2006). Transcriptional regulatory networks in cellular responses and tolerance to dehydration and cold stresses.. Annual Review of Plant Biology.

[10] Zhu JK (2002). Salt and drought stress signal transduction in plants.. Annu Rev Plant Biol.

[11] Pandey S, Chen JG, Jones AM, Assmann SM (2006). G-protein complex mutants are hypersensitive to abscisic acid regulation of germination and postgermination development.. Plant Physiol.

[12] Hegedus D, Yu M, Baldwin D, Gruber M, Sharpe A (2003). Molecular characterization of Brassica napus NAC domain transcriptional activators induced in response to biotic and abiotic stress.. Plant Mol Biol.

[13] Leung J, Bouvier-Durand M, Morris PC, Guerrier D, Chefdor F (1994). Arabidopsis ABA response gene *ABI1*: features of a calcium-modulated protein phosphatase.. Science.

[14] Rodriguez PL, Benning G, Grill E (1998). ABI2, a second protein phosphatase 2C involved in abscisic acid signal transduction in Arabidopsis.. Febs Letters.

[15] Allen GJ, Kuchitsu K, Chu SP, Murata Y, Schroeder JI (1999). Arabidopsis *abi1-1* and *abi2-1* phosphatase mutations reduce abscisic acid-induced cytoplasmic calcium rises in guard cells.. Plant Cell.

[16] Giraudat J, Hauge BM, Valon C, Smalle J, Parcy F (1992). Isolation of the Arabidopsis *ABI3* gene by positional cloning.. Plant Cell.

[17] Finkelstein RR, Wang ML, Lynch TJ, Rao S, Goodman HM (1998). The Arabidopsis abscisic acid response locus ABI4 encodes an APETALA 2 domain protein.. Plant Cell.

[18] Finkelstein RR, Lynch TJ (2000). The Arabidopsis abscisic acid response gene *ABI5* encodes a basic leucine zipper transcription factor.. Plant Cell.

[19] Porta H, Rocha-Sosa M (2002). Plant lipoxygenases. Physiological and molecular features.. Plant Physiol.

[20] Feussner I, Kindl H (1992). A lipoxygenase is the main lipid body protein in cucumber and soybean cotyledons during the stage of triglyceride mobilization.. FEBS Lett.

[21] Terp N, Gobel C, Brandt A, Feussner I (2006). Lipoxygenases during Brassica napus seed germination.. Phytochemistry.

[22] Melan MA, Enriquez A, Peterman TK (1994). The LOX1 Gene of Arabidopsis Is Temporally and Spatially Regulated in Germinating Seedlings.. Plant Physiol.

[23] Park TK, Holland MA, Laskey JG, Polacco JC (1994). Germination-associated lipoxygenase transcripts persist in maturing soybean plants and are induced by jasmonate.. Plant Science.

[24] Porta H, Rueda-Benitez P, Campos F, Colmenero-Flores JM, Colorado JM (1999). Analysis of lipoxygenase mRNA accumulation in the common bean (*Phaseolus vulgaris L.*) during development and under stress conditions.. Plant Cell Physiol.

[25] Arteaga S, Andrade-Cetto A, Cardenas R (2005). Larrea tridentata (*Creosote bush*), an abundant plant of Mexican and US-American deserts and its metabolite nordihydroguaiaretic acid.. Journal of Ethnopharmacology.

[26] Floriano-Sanchez E, Villanueva C, Medina-Campos ON, Rocha D, Sanchez-Gonzalez DJ (2006). Nordihydroguaiaretic acid is a potent in vitro scavenger of peroxynitrite, singlet oxygen, hydroxyl radical, superoxide anion and hypochlorous acid and prevents in vivo ozone-induced tyrosine nitration in lungs.. Free Radic Res.

[27] Van Wauwe J, Goossens J (1983). Effects of antioxidants on cyclooxygenase and lipoxygenase activities in intact human platelets: comparison with indomethacin and ETYA.. Prostaglandins.

[28] Creelman RA, Bell E, Mullet JE (1992). Involvement of a Lipoxygenase-Like Enzyme in Abscisic Acid Biosynthesis.. Plant Physiol.

[29] Ren HB, Gao ZH, Chen L, Wei KF, Liu J (2007). Dynamic analysis of ABA accumulation in relation to the rate of ABA catabolism in maize tissues under water deficit.. Journal of Experimental Botany.

[30] Kirik V, Bouyer D, Schobinger U, Bechtold N, Herzog M (2001). CPR5 is involved in cell proliferation and cell death control and encodes a novel transmembrane protein.. Curr Biol.

[31] Yoshida S, Ito M, Nishida I, Watanabe A (2002). Identification of a novel gene *HYS1/CPR5* that has a repressive role in the induction of leaf senescence and pathogen-defence responses in Arabidopsis thaliana.. Plant J.

[32] Bowling SA, Clarke JD, Liu Y, Klessig DF, Dong X (1997). The cpr5 mutant of Arabidopsis expresses both NPR1-dependent and NPR1-independent resistance.. Plant Cell.

[33] Boch J, Verbsky ML, Robertson TL, Larkin JC, Kunkel BN (1998). Analysis of resistance gene-mediated defense responses in Arabidopsis thaliana plants carrying a mutation in CPR5.. Molecular Plant-Microbe Interactions.

[34] Brininstool G, Kasili R, Simmons A, Kirik V, Hülskamp M (2008). *Constitutive Expressor of Pathogenesis-Related Genes 5* affects cell wall biogenesis and trichome development.. BMC Plant Biol.

[35] Jing HC, Dijkwel PP (2008). CPR5: A Jack of all trades in plants.. Plant Signal Behav.

[36] Clough SJ, Bent AF (1998). Floral dip: a simplified method for Agrobacterium-mediated transformation of Arabidopsis thaliana.. Plant J.

[37] Ichida F, Uese K, Hashimoto I, Hamamichi Y, Tsubata S (1997). Acute effect of oral prostacyclin and inhaled nitric oxide on pulmonary hypertension in children.. J Cardiol.

[38] Giraudat J (1995). Abscisic-Acid Signaling.. Current Opinion in Cell Biology.

[39] Turner JG, Ellis C, Devoto A (2002). The jasmonate signal pathway.. Plant Cell.

[40] Dekkers BJ, Schuurmans JA, Smeekens SC (2004). Glucose delays seed germination in Arabidopsis thaliana.. Planta.

[41] Chen Y, Ji F, Xie H, Liang J, Zhang J (2006). The regulator of G-protein signaling proteins involved in sugar and abscisic acid signaling in Arabidopsis seed germination.. Plant Physiol.

[42] Shang Y, Yan L, Liu Z, Cao Z (2010). The Mg-Chelatase H subunit of *Arabidopsis* sntagonizes a group of WRKY transcription repressors to relieve ABA-responsive genes of Inhibition.. Plant Cell.

[43] Schroeder JI, Allen GJ, Hugouvieux V, Kwak JM, Waner D (2001). Guard Cell Signal Transduction.. Annu Rev Plant Physiol Plant Mol Biol.

[44] Sirichandra C, Wasilewska A, Vlad F, Valon C, Leung J (2009). The guard cell as a single-cell model towards understanding drought tolerance and abscisic acid action.. J Exp Bot.

[45] Hetherington AM (2001). Guard cell signaling.. Cell.

[46] Bu Q, Li H, Zhao Q, Jiang H, Zhai Q (2009). The Arabidopsis RING finger E3 ligase RHA2a is a novel positive regulator of abscisic acid signaling during seed germination and early seedling development.. Plant Physiol.

[47] Ramirez V, Coego A, Lopez A, Agorio A, Flors V (2009). Drought tolerance in Arabidopsis is controlled by the OCP3 disease resistance regulator.. Plant J.

[48] Bokoch GM, Reed PW (1981). Effect of various lipoxygenase metabolites of arachidonic acid on degranulation of polymorphonuclear leukocytes.. J Biol Chem.

[49] Ren HB, Fan YJ, Gao ZH, Wei KF, Li GF (2007). Roles of a sustained activation of NCED3 and the synergistic regulation of ABA biosynthesis and catabolism in ABA signal production in Arabidopsis.. Chinese Science Bulletin.

[50] Zhu GH, Ye NH, Zhang JH (2009). Glucose-Induced Delay of Seed Germination in Rice is mediated by the Suppression of ABA Catabolism rather than an Enhancement of ABA Biosynthesis.. Plant and Cell Physiology.

[51] Brash AR (1999). Lipoxygenases: Occurrence, functions, catalysis, and acquisition of substrate.. Journal of Biological Chemistry.

[52] Feussner I, Kuhn H, Wasternack C (2001). Lipoxygenase-dependent degradation of storage lipids.. Trends Plant Sci.

[53] Kato T, Ohta H, Tanaka K, Shibata D (1992). Appearance of New Lipoxygenases in Soybean Cotyledons after Germination and Evidence for Expression of a Major New Lipoxygenase Gene.. Plant Physiol.

[54] Holtman WL, Vredenbregt-Heistek JC, Schmitt NF, Feussner I (1997). Lipoxygenase-2 oxygenates storage lipids in embryos of germinating barley.. Eur J Biochem.

[55] Ohta H, Ida S, Mikami B, Morita Y (1986). Changes in lipoxygenase components of rice seedlings during germination.. Plant and Cell Physiology.

[56] Vick BA, Zimmerman DC (1976). Lipoxygenase and Hydroperoxide Lyase in Germinating Watermelon Seedlings.. Plant Physiol.

[57] Feussner I, Wasternack C (2002). The lipoxygenase pathway.. Annu Rev Plant Biol.

[58] Hamberg M, Ponce de Leon I, Rodriguez MJ, Castresana C (2005). Alpha-dioxygenases.. Biochem Biophys Res Commun.

[59] Farmer EE (1994). Fatty acid signalling in plants and their associated microorganisms.. Plant Mol Biol.

[60] Creelman RA, Mullet JE (1997). Biosynthesis and Action of Jasmonates in Plants.. Annu Rev Plant Physiol Plant Mol Biol.

