# Supplementary figures and images for: Arabidopsis CPR5 Independently Regulates Seed Germination and Postgermination Arrest of Development through LOX Pathway and ABA Signaling
Source: PLoS One. 2011 Apr 27;6(4):e19406. doi: 10.1371/journal.pone.0019406 (PMC3083440; doi:10.1371/journal.pone.0019406)

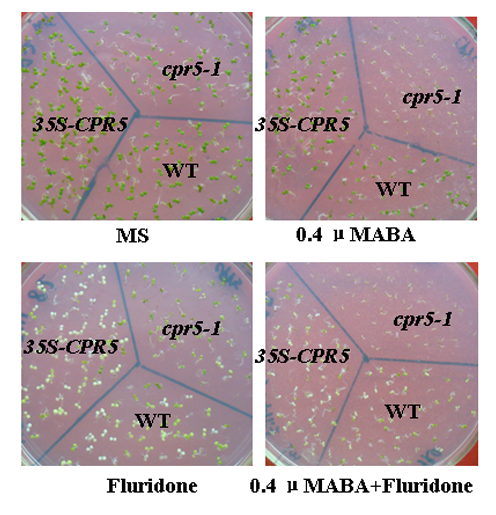

Supplement: Figure S1 — The influence of fluridone on ABA sensitivity of cpr5-1 and 35S-CPR5 plants. Matched seed lots were pretreated with deionized water or 100 mM fluridone for 24 h at 4°C before being placed at 22°C for germination. Seeds were germinated on MS and MS medium containing 0.4 µM ABA, and grown for 5 days. (TIF) [file pone.0019406.s001.tif]

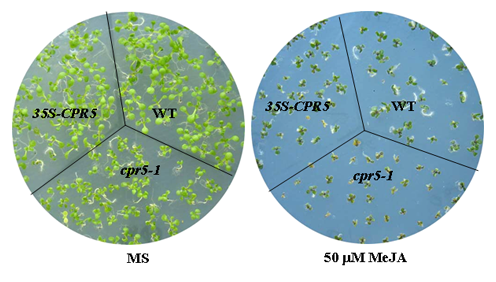

Supplement: Figure S2 — MeJA response analysis of cpr5-1 and 35S-CPR5 plants. Seeds of wild type, cpr5, 35S-CPR5 plants were germinated and growth for 12 days on MS medium containing 0 µM and 50 µM MeJA. (TIF) [file pone.0019406.s002.tif]

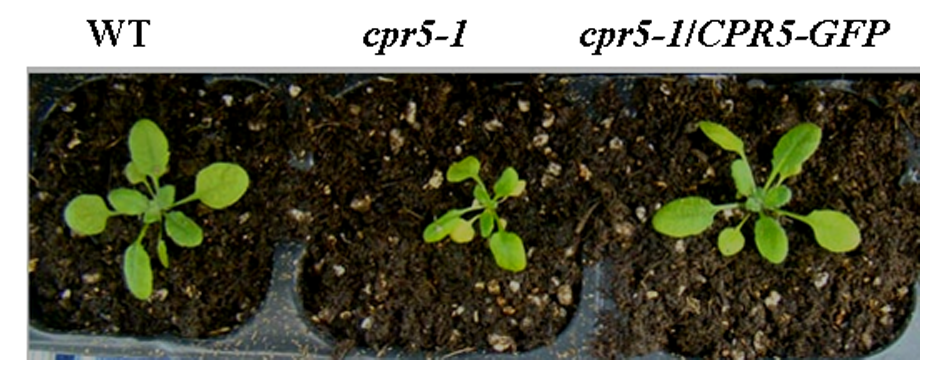

Supplement: Figure S3 — 35S-CPR5-EGFP fusion transgenic plant complements the phenotype of cpr5-1 mutant. Two-week-old seedlings of 35S-CPR5-EGFP in cpr5-1 showing the phenotype of the wild-type. (TIF) [file pone.0019406.s003.tif]
