# Supplementary material for: Arabidopsis CPR5 Independently Regulates Seed Germination and Postgermination Arrest of Development through LOX Pathway and ABA Signaling
Source: PLoS One. 2011 Apr 27;6(4):e19406. doi: 10.1371/journal.pone.0019406 (PMC3083440; doi:10.1371/journal.pone.0019406)
Supplement: Table S1 — Primers used in this study. (DOC) [file pone.0019406.s004.doc]

**Table S1**. Gene-specific primer sequences used to detect genes expression by Real time-PCR

| Genes | TAIR locus | Primers |
| --- | --- | --- |
| *CPR5* F | At5g64930 | GATTGGCTCCTCGTAAGTGTCTTC |
| *CPR5* R | At5g64930 | TCTCCTCGGATTGGTCGCATAC |
| *ABI1* F | At4g26080 | GTGGAAGAAGACCTGAGATGGAAG |
| *ABI1* R | At4g26080 | AAGAAATGAGCGGCGGATTGAG |
| *AtPP2CA* F | At3g11410 | GATATGATGGACGGCGTTGAAGG |
| *AtPP2CA* R | At3g11410 | TGATGTGTTTGACAAGAGCAGAGG |
| *ABI2* F | At5g57050 | GATGATGAAGCGGCGAGGATAG |
| *ABI2* R | At5g57050 | TGCGAGAACACCAAATACACGAG |
| *ABI5* F | At2g36270 | AATGGAGGAGGTGGTGGTGAG |
| *ABI5* R | At2g36270 | CTGCTGCTGCTTGTTGTTGATTG |
| *ABF3* F | At4g34000 | TGTCTCACGGCTTTGGATTTGG |
| *ABF3* R | At4g34000 | GTTGGCTGCTGCTCACTCAC |
| *ABF4* F | At3g19290 | ACTTGTCGTCTCTCCCTGTAGC |
| *ABF4* R | At3g19290 | GACCACCACCTCCTAAGTTGTTG |
| *RD29A* F | At5g52310 | GTGACGACGAAGTTACCTATCTCC |
| *RD29A* R | At5g52310 | TCTCCGCCACATAATCTCTACCC |
| *RD29B* F | AT5G52300 | CAGACAGAGGAGAGAGCAGAGAG |
| *RD29B* R | AT5G52300 | CTTCACCACCAGGAGCAAACG |
| *RD22* F | At5g25610 | GCCACAAGGGAAGACCGATTTAC |
| *RD22* R | At5g25610 | TGCCTCCGTAACCATCCTCAG |
| *RAB18* F | At5g66400 | GTCTTACTGCTGAAGGTTCGTCTG |
| *RAB18* R | At5g66400 | ATCCAAGATGCTGCGGTTTAGG |
| *UBQ10* F | At4g05320 | CGGAAAGCAGTTGGAGGATGG |
| *UBQ10* R | At4g05320 | CGGAGCCTGAGAACAAGATGAAG |
